# Risk of cervical high-grade squamous intraepithelial neoplasia in cytologic negative and persistently high-risk human papillomavirus positive patients according to genotypes: a retrospective single center analysis

**DOI:** 10.1186/s12879-024-09449-z

**Published:** 2024-06-04

**Authors:** Barbara Kipp, Elena Ulrich, Carmen van Meegen, Thomas Schwenzer

**Affiliations:** 1 Department of Gynecology and Obstetrics, Klinikum Dortmund gGmbH, Beurhausstr. 40, 44137 Dortmund, Germany; 2 Department of Health, Witten/Herdecke University, Witten, Germany; 3 Center for Higher Education, Statistical Consulting and Analysis, TU Dortmund University, Vogelpothsweg 78, 44221 Dortmund, Germany

**Keywords:** High-risk human papillomavirus, High-grade cervical intraepithelial neoplasia, Genotyping, Negative cytology, Colposcopy, oKFE-RL, long-term risk

## Abstract

In January 2020, a different cervical cancer screening program started in Germany. Women above the age of 35 are recommended to have a combined HPV and cytology swab every three years. Showing persistent high-risk human papillomavirus (hrHPV), cytologic negative cervical samples at baseline and after 12 months, patients are referred to colposcopy. Entailing considerable additional workload due to the required colposcopies, we analyzed the risk of high-grade cervical intraepithelial neoplasia (CIN 3) in cytologic negative and persistent hrHPV women according to their hrHPV genotypes.

**Methods **In this single center retrospective study, patients with persistent hrHPV, cytology negative cervical samples from our certified Colposcopy Unit in 2020 and 2021 were analyzed. Patient demographics, hrHPV types, biopsy rates and histological reports were collected.

**Results **During the study, 69 patients were enrolled. Most frequent hrHPV genotypes were: hrHPV other 72.5%; HPV 16, 20.3% and HPV 18, 7.2%. Colposcopy showed no or minor changes in 92.7% and major changes in 7.2%. CIN 3 was found in 7 patients (10.1%). Prevalence of CIN 3 by hrHPV genotypes was 27.3% for HPV16, 20.0% for HPV18 and 7.1% for HPVO. A statistically significant dependency between hrHPV and cervical intraepithelial neoplasia was demonstrated (*p* = 0.048).

**Conclusion **Within this single center study of persistent hrHPV, cytologic negative samples, patients with HPV 16 were more likely to have high-grade disease compared to other hrHPV subtypes. Larger prospective randomized trials are needed to substantiate our results and obtain adjusted cervical cancer screening time intervals according to the hrHPV genotypes.

## Introduction

Cervical cancer is the fourth most frequently diagnosed cancer worldwide and the second leading cause of cancer death in women with a cervix [1]. In Germany the prevalence of cervical cancer and precancerous lesions has declined over the last 30 years due to opportunistic screening and increased vaccination rates against high-risk human papillomavirus (hrHPV) types [2–4]. Nevertheless, the focus on prevention and early diagnosis of cervical cancer remains crucial. It is widely acknowledged that high-grade cervical intraepithelial neoplasia and invasive cervical cancers are caused by persistent hrHPV infections [5]. The human papillomavirus (HPV) genotypes 16 and 18 are responsible for more than 75% of cervical carcinomas and the precursor lesions. The risk of persistence and neoplastic progression to high-grade cervical intraepithelial neoplasia and cervical cancer differs markedly by HPV genotype. Although several HPV types have been characterised as high-risk HPV with carcinogenic potential, they do not appear to display a comparable carcinogenicity. Infection with HPV 16 has the greatest tendency to persist and the highest probability for causing neoplastic progression, followed by infection with HPV 18, HPV 31 and HPV 33 [6, 7]. These facts indicate the potential value of additional genotyping in cervical cancer screening. In Germany, cervical cancer screening is conducted in accordance with the 2020 oKFE-RL Cancer Screening Guidelines, which recommend co-testing for cytology and HPV DNA in patients aged 35 years and older. Joint cytology and HPV screening at 3-year intervals appears to constitute a low risk of CIN 3 in patients who were negative for both HPV and cytology testing at baseline, and they are referred to repeating co-testing every 3 years. Patients with normal Papanicolaou (PAP) smear (PAP I) and the detection of high-risk HPV DNA at baseline and one year thereafter are referred to colposcopy due to the increased risk of developing CIN 3.

The introduction of this new approach to cervical cancer screening has led to an increase in the number of colposcopies required in cytology- negative patients.The aim of this study was to analyse the risk of high-grade cervical intraepithelial neoplasia in cytologically negative and persistent hrHPV patients according to their HPV genotypes.

## Methods

This retrospective study was conducted in the dysplasia unit of the Klinikum Dortmund gGmbH. Only patients above the age of 35 with negative cytology and persistent positive hrHPV for at least 12 months met the inclusion criteria and were identified through the institutional dysplasia database. The results of cytological examinations and HPV tests were obtained from patients` gynaecologists. Cytology was performed in accordance with standard procedure. The specific HPV tests chosen were not explicitly specified. The results differentiated between HPV 16 or HPV 18 or HPVO (= others) positivity. The subgroup “others” comprises the WHO defined high risk subtypes except 16 and 18 (31,33,35,39,45,51,52,56,58,59,66). Colposcopic examination was conducted by two senior consultants. Histopathological findings and detailed information were extracted from the database of the Institute of Pathology. Patient demographics, HPV genotype, colposcopic assessment, biopsy rates and histologic diagnosis were recorded.

A comprehensive descriptive and exploratory analysis of the data was performed, including frequencies and percentages. Fisher´s Exact Test was applied to test the global hypothesis of an association between HPV genotypes and histology diagnoses [8]. Furthermore, the influence of hrHPV genotype on the categories of cervical intraepithelial neoplasia was examined using a proportional odds model that also accounted for covariates such as colposcopy results, transformation zone and age [9]. For all statistical tests, a level of significance of 0.05 was considered. All statistical analyses were conducted using the statistical software R (version 4.3.0) [10]. In addition, the R package ordinal was used for modelling [11].

The study was approved by the ethics committee of the university Witten/Herdecke (S-03/2023). It was performed in accordance with the principles of good clinical practice and the Declaration of Helsinki. As the study involved retrospective evaluation of routine data, the Ethics committee granted exemption from the requirement of informed consent.

## Results

**Fig. 1:**
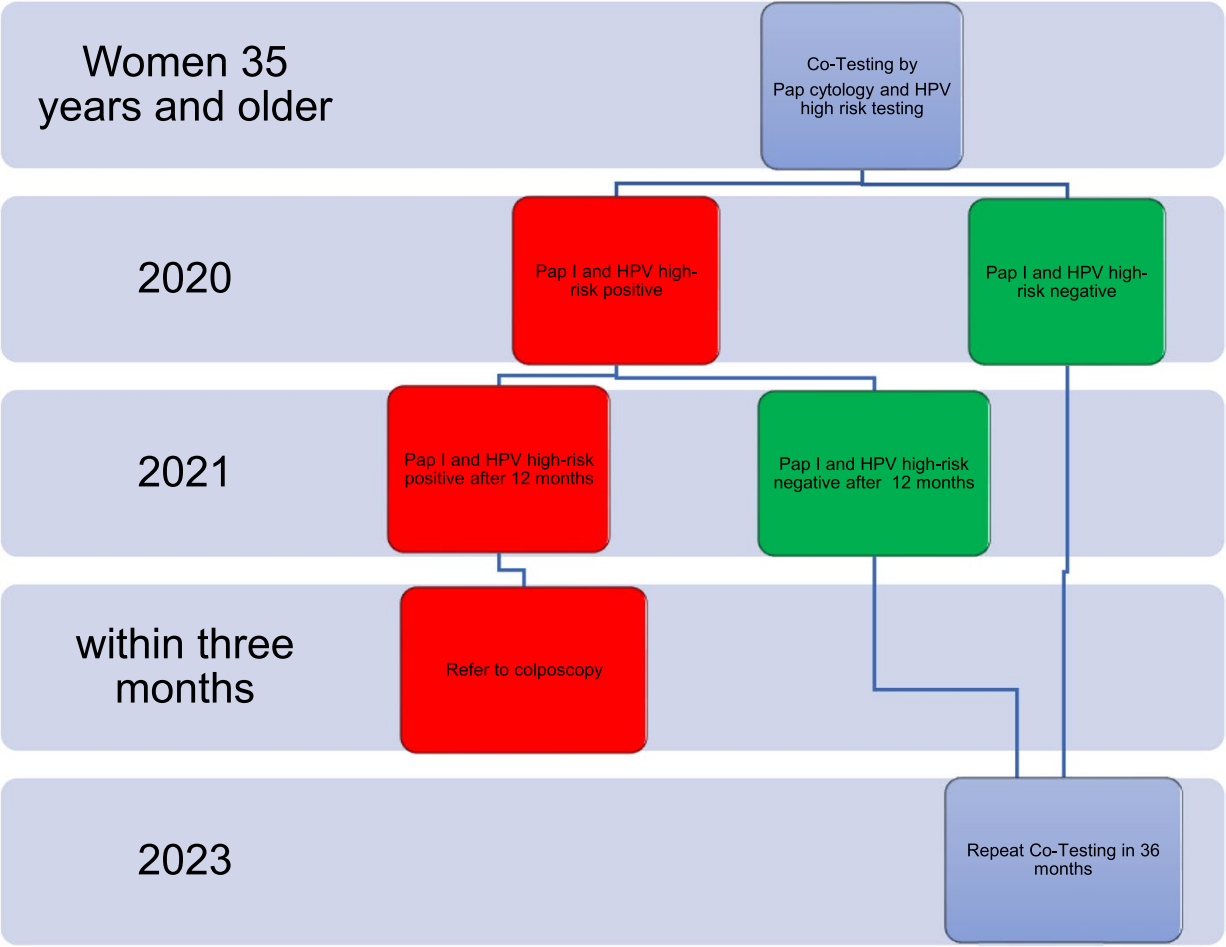
oKFE-RL´s new referral pathway

Following referral with persistently positive hrHPV, cytologically negative cervical samples, 69 patients were examined as new patients in the certified Colposcopy Unit between 1/1/2020 and 12/31/2021 according to the oKFE-RL´s new referral pathway (Fig. 1).

### Patient characteristics

Median age at referral was 51 years (range: 35–82 years). The grouped age frequencies are listed in Table 1. The most frequent hrHPV was HPVO with 72.5% (*n* = 50), followed by HPV 16 with 20.3% (*n* = 14) and HPV 18 with 7.2% (*n* = 5). Transformation zone (TZ) 3 was the predominant zone in 68.1% of patients (*n* = 47), in contrast to TZ 1 and 2 in 31.9% of patients (*n* = 22). In 84.1% of patients (*n* = 58), a target biopsy or endocervical curettage (ECC) was performed during the colposcopy assessment.

**Table 1:** Demographics

		*n*	*%*
**Age group**	*[35,45)*	18	26.1
	*[45,55)*	26	37.7
	*[55,65)*	18	26.1
	*[65,85]*	7	10.1
**HPV genotype**	*type 16*	14	20.0
	*type 18*	5	7.2
	*others*	50	72.5
**Transformation zone**	*1/2*	22	31.9
	*3*	47	68.1
**Biopsy**	*none*	11	15.9
	*endocervical*	36	52.2
	*ectocervical*	18	26.1
	*both*	4	5.8

### Outcomes from colposcopy

Adequate colposcopy assessment was performed by the two senior consultants in all examined patients. 56.5% (*n* = 39) showed normal colposcopy, minor and major changes were detected in 36.2% (*n* = 23) and 7.2% (*n* = 5), respectively. In the subgroup of HPV 16 positive patients, normal colposcopy, minor and major changes were found in 50.0%, 35.7% and 14.3%, respectively. In comparison, in HPVO positive patients, normal colposcopy was predominant with 62.0%, followed by 34.0% with minor and 4.0% with major changes. In the subgroup of HPV 18 positive patients, only one colposcopy assessment was normal (20.0%), three patients (60.0%) showed minor, and one patient major changes (20.0%).

Thirty-two biopsies showed normal histology. In this group, 14 patients (43.8%) exhibited minor and two patients (6.2%) exhibited major changes in colposcopy, whereas eight out of ten patients with CIN 2 had normal colposcopy assessments. This discrepancy can be attributed to the fact that the majority of patients with CIN 2 had TZ 3 (90.0%), which represents one of the most challenging aspects of colposcopy. Only one patient with histological confirmation of CIN 3 exhibited normal colposcopy findings (14.3%). For CIN 3, the majority of patients demonstrated minor changes (57.1%).

### Histological diagnosis and HPV genotype

**Fig. 2:**
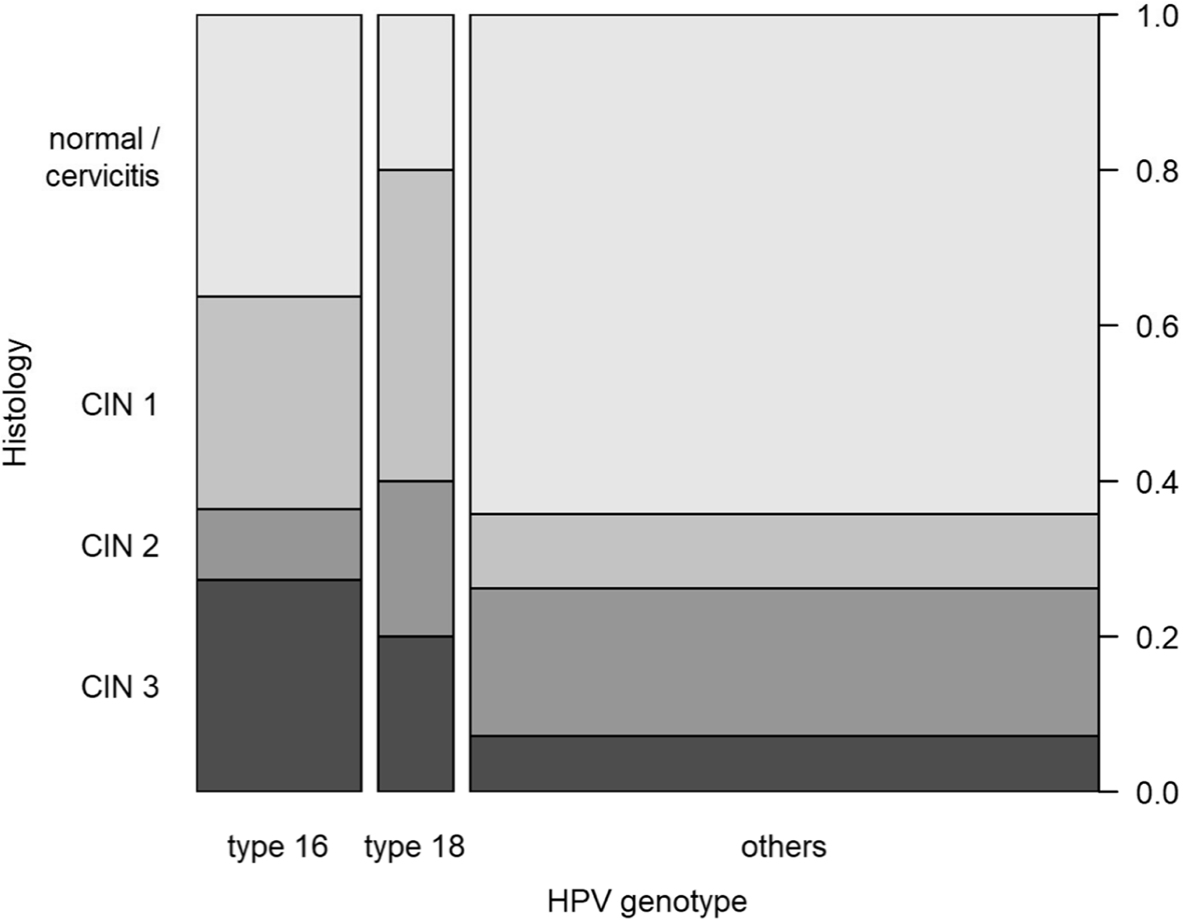
Mosaicplot of histological diagnosis and hrHPV types

A total of 11 patients without biopsies were excluded from the subsequent statistical analysis. The remaining 58 patients are the focus of our discussion. During the study period, no cancers were detected in patients referred with persistently positive hrHPV samples whose cytology was negative. In total, 15.5% of referrals were diagnosed with CIN I (*n* = 9), 17.2% with CIN 2 (*n* = 10), and 12.1% with CIN 3 (*n* = 7). Furthermore, the histologic results were analysed in the context of hrHPV genotypes. Table 2 and the corresponding mosaicplot in Fig. 2 illustrate the frequency distribution of histology findings for the biopsied patients, stratified by HVP 16, 18, and HPVO.

**Table 2:** Frequency table of Histology and HPV

	**Histology**				
**HPV genotype**	*normal / cervicitis*	*CIN 1*	*CIN 2*	*CIN 3*	Sum
*type 16*	4 (36.4%)	3 (27.3%)	1 (9.1%)	3 (27.3%)	11 (100%)
*type 18*	1 (20.0%)	2 (40.0%)	1 (20.0%)	1 (20.0%)	5 (100%)
*others*	27 (64.3%)	4 (9.5%)	8 (19.0%)	3 (7.1%)	42 (100%)
Sum	32 (55.2%)	9 (15.5%)	10 (17.2%)	7 (12.1%)	58 (100%)

Among patients with HPV 16, CIN 2 was observed only once, whereas CIN 1 and 3 were diagnosed in 27.3% (*n* = 3) of cases, respectively. In HPV 18 positive patients, CIN 1 was found in 40.0% of cases (*n* = 2). Other CIN stages were observed only once each (20.0% each). In patients with HPVO the prevalence of CIN 3 was 7.1% (*n* = 3). Normal histology was most frequently observed within HPVO (64.3%). Based on Table 2 Fisher’s Exact Test demonstrated a statistically significant dependency between hrHPV genotypes and cervical intraepithelial neoplasia (*p* = 0.048).

To quantify the relationship between histologic findings and hrHPV genotypes, a proportional odds model was developed to consider colposcopy results, transformation zone, and age.

The proportional odds model did not yield statistically significant results, which is likely due to the limited sample size in this study.

## Discussion

In recent years, it has been established that high-grade cervical neoplasia and invasive cervical cancers are caused by persistent infection with hrHPV [6, 12]. Primary HPV screening is significantly more sensitive for high-grade disease prediction than cytology alone [13]. Long-term data from HPV screening trials indicate a low risk of high-grade CIN in HPV negative and cytologically negative patients [14, 15]. Conversely, in patients with negative cytology and persistent hrHPV infection, high-grade CIN and cervical cancer rates are notably higher [14].

Following the implementation of HPV-based cervical cancer screening in several countries (Australia, Italy, Netherlands, New Zealand, Sweden or the UK) for an extended period, the introduction of HPV- and cytology-based screening in Germany commenced in 2020. The growing number of colposcopies resulting from the implementation of the new co-testing (cytology and test for high-risk HPV), has been the subject of considerable debate, as under the new national guidelines hrHPV positive patients with normal cytology are now additionally referred for colposcopy [16]. At our institution, we observed a noteworthy increase of 30% in the number of colposcopies completed.

In this study,we present data on the risk of CIN 3 according to the hrHPV genotype in cytologically negative patients. Regarding the quality of colposcopy, only adequate colposcopic settings were performed by two senior consultants. The rate of inadequate colposcopy in patients with persistent hrHPV varies in literature between 0.59% [17], and 4.8% [13].

In our sample, transformation zone 3 (TZ 3) was predominant with 68.1% while transformation zone 1/2 occurred in 31.9%. This is likely due to the median age of 51 years in our group. Furthermore, the new screening is intended for patients over the age of 35. These findings are consistent with a German cohort analysis which demonstrated TZ 3 in 62.6% and TZ 1/2 in 37.5%, respectively [17].

The colposcopic appearance is thought to depend on the HPV high-risk subtype. In the case of HPVO, 62.0% of patients exhibited normal colposcopy, while in HPV 16, minor and major changes were observed in 50.0%. Jeronimo et al. 2007 reported increased detection of abnormalities in colposcopy in the presence of HPV 16, while van der Marel et al. 2014 denied any relationship between colposcopic characteristics and HPV 16 [18, 19]. The question of whether certain hrHPV genotypes have an impact on colposcopic appearance remains open.

The biopsy rate in our study was 84.1%. which is considerably higher than the rates reported in the literature. A review of European studies that investigated the efficacy of HPV-based screening revealed biopsy rates ranging from 2 to 11%. In contrast a biopsy rate of 31% was found in a retrospective cohort study in England [13, 15].

No cases of invasive cancer were identified in our cohort, but 15.5%, 17.2%, and 12.1% of participants exhibited CIN 1, CIN 2 and CIN 3,respectively. The increased detection of CIN 1 and CIN 2 may give rise to concerns regarding the potential for overtreatment of early lesions, as CIN 1 and CIN 2 may regress spontaneously in 60% and 55% of cases, respectively [20]. The regression rate of CIN 1 and 2 was significantly higher in hrHPV negative patients younger than 30 years.The overall progression to invasive cancer was less than 0.5% [20]. Therefore, conservative treatment might be considered. The incidence of CIN 3 (12.1%) observed in our study is consistent with other authors who found 13.0% of CIN 2 + in their register analysis of 1139 cytologic negative, hrHPV positive patients [21]. A focus on the hrHPV genotypes revealed that normal histology was the predominant finding in HPVO (64.3%), followed by HPV 16 (36.4%) and HPV 18 (20.0%). In contrast CIN 3 was mainly observed in HPV 16 (27.3%), followed by HPV 18 (20.0%) and HPVO (7.1%).

Our findings are supported by a danish cohort study.This study demonstrated that CIN 3 occurs in 26.7% of cases with HPV 16 and 19.1% of cases with HPV 18 [6]. The HPV testing strategy employed in this study included a differential testing of 13 high-risk HPV subtypes. The data from this study group also revealed that HPV 31 and 33 exhibited noteworthy carcinogenic potential (14.3%, and 14.9%, respectively) [6]. All remaining hrHPV subtypes were summarized as HPVO with an estimated probability of CIN 3 of 6.0%. Our study revealed a higher risk of developing high-grade cervical neoplasia in patients with HPV 16 compared to other HPV types. This is consistent with other authors who have demonstrated that HPV 16 has the highest oncogenic potential and the highest likelihood of developing CIN 3, [6, 22–24].

The limitation of our study results from the retrospective data collection and the small number of patients in a single centre dysplasia unit. We only obtained both the results of cytology and the HPV-testing from the gynaecologists. Cytology was performed conventionally as Thin prep is not supported by the healthcare system. The HPV test chosen was not explicitly specified. The results were divided into HPV 16 or HPV 18 or HPV others. HPV- “Others” is a generally accepted summary of the remaining WHO recognized subtypes (31,33,35,39,45,51,52,56,58,59,66). A complete typification was not available in our study.

Our retrospective data suggest that extended screening intervals could be possible without risk in cytologic negative hrHPV positive patients that is not 16 or 18 as the risk of CIN was relevantly lower in our HPVO subgroup.

For future investigations prospective data collection containing complete HPV genotyping will be of great interest. The results might exactly demonstrate the statistical risk of developing cervical precusor lesions, CIN 3 in particular, according to hrHPV genotype and will possibly have an impact on screening intervals without comprising patient safety.

## Data Availability

The datasets generated or analyzed during the current study are not publicly available but are available from the correspondig author upon reasonable request.

## Abbreviations

HPV: Human papilloma virus
hrHPV: High-risk human papillomavirus
DNA: Desoxyribonucleic acid
CIN: Cervical intraepithelial neoplasia
PAP: Papanicolaou
HPVO: Human papillomavirus type others (high-risk, not 16,18)
WHO: World health organization
TZ: Transformation zone
ECC: Endocervical curettage
